# Qualitative development and content validation of the “SPART” model; a focused ethnography study of observable diagnostic and therapeutic activities in the emergency medical services care process

**DOI:** 10.1186/s12873-021-00526-z

**Published:** 2021-11-13

**Authors:** Bert Dercksen, Michel M. R. F. Struys, Fokie Cnossen, Wolter Paans

**Affiliations:** 1 University Medical Centre Groningen-department of anesthesiology/HEMS, Hanzeplein 1, 9713GZ Gn Groningen, The Netherlands; 2 UMCG Ambulance Care, Vriezerweg 10, 9482TB Dr Tynaarlo, The Netherlands; 3 Faculty of Medical Sciences, University of Groningen, Antonius Deusinglaan 1, 9713AV Groningen, The Netherlands; 4 Faculty of Science and Engineering Artificial Intelligence, Bernoulli Institute, University of Groningen, Nijenborgh 9, 9747AG Groningen, The Netherlands; 5 Hanze University of Applied Sciences-Nursing Diagnostics and Centre of Expertise Healthy Ageing, Zernikelaan 6, 9747AA Groningen, the Netherlands

**Keywords:** SPART, Emergency medical services, EMS care process, Paramedic process, Ambulances

## Abstract

**Background:**

Clinical reasoning is a crucial task within the Emergency Medical Services (EMS) care process. Both contextual and cognitive factors make the task susceptible to errors. Understanding the EMS care process’ structure could help identify and address issues that interfere with clinical reasoning. The EMS care process is complex and only basically described.

In this research, we aimed to define the different phases of the process and develop an overarching model that can help detect and correct potential error sources, improve clinical reasoning and optimize patient care.

**Methods:**

We conducted a focused ethnography study utilizing non-participant video observations of real-life EMS deployments combined with thematic analysis of peer interviews.

After an initial qualitative analysis of 7 video observations, we formulated a tentative conceptual model of the EMS care process. To test and refine this model, we carried out a qualitative, thematic analysis of 28 video-recorded cases. We validated the resulting model by evaluating its recognizability with a peer content analysis utilizing semi-structured interviews.

**Results:**

Based on real-life observations, we were able to define and validate a model covering the distinct phases of an EMS deployment. We have introduced the acronym “SPART” to describe ten different phases: Start, Situation, Prologue, Presentation, Anamnesis, Assessment, Reasoning, Resolution, Treatment, and Transfer.

**Conclusions:**

The “SPART” model describes the EMS care process and helps to understand it. We expect it to facilitate identifying and addressing factors that influence both the care process and the clinical reasoning task embedded in this process.

**Supplementary Information:**

The online version contains supplementary material available at 10.1186/s12873-021-00526-z.

## Introduction

### Background

In the Emergency Medical Services, like in general medicine, the clinical reasoning process is regarded as one of the main pillars on which care provision is built. A significant body of evidence indicates that the process is prone to flaws resulting in diagnostic errors. Many of these mistakes are caused by cognitive reasoning errors [1–4], are related to the high context-dependency [5] of the clinical reasoning process, or are associated with the intricate interplay between these factors [1, 6, 7]. Compared to medicine in a general hospital, EMS care is more prone to diagnostic errors because of time pressure, lack of available information, interruptions, and distractions. The conditions under which the work must be done are very similar to the work in an emergency department. Carter and Thomson [8] proposed a “paramedic process” model because a specific model adapted to the EMS practice’s professional status did not exist. The theoretically developed model described the EMS workflow in specific detail. Their model was a modification of the nursing process [9]. The traditional nursing process consists of 5 sequential steps: assessment, diagnosis, planning, implementation, and evaluation. Carter and Thomson expanded this concept with additional steps to bring it more into line with EMS practice. The proposed paramedic process model considers the specific nature of the pre-hospital medical practice. They defined the steps: dispatch considerations, scene assessment, first impressions, patient history, physical examination, clinical decision making, interventions, re-evaluation, transport decision, hand-over/documentation and reflection. However, to our knowledge, this theoretical model was never tested or validated. Therefore, we conceived a study that could map the complete EMS care process based on real-life observations.

Understanding the EMS care process structure could help identify and address the factors that interfere with the process itself and the embedded clinical reasoning task.

### Aim

This study aims to clarify the EMS care process and develop an overarching process model by studying real-life EMS deployments.

## Methods

### Study design and setting

We used a mixed-method study design based on focused ethnography principles consisting of non-participant real-life video observations of EMS deployments combined with a peer content analysis by semi-structured interviews.

Focused ethnography involves collecting data focusing on a specific aspect of a community’s activity within a limited period of time [10, 11]. The non-participant observation method [12] implies no interaction between the researcher and the observed participant during the observation. We carried out this research amongst EMS clinicians of three different Dutch EMS organizations. These three organizations were selected for practical reasons. The three EMS organizations were considered representative for the Dutch system because of the standardization and uniformity, regulated by law and regulations, of all EMS organizations operating in the country. The real-life video observations were carried out in one of these three organizations. The semi-structured interviews conducted in de validation phase of the model were executed in the remaining two organizations. Participants in the latter were unaware of the real-life observational study conducted in the first EMS organization.

### EMS in the Netherlands

Ambulance care in the Netherlands is carried out by mixed teams. An Advanced Life Support ambulance is operated by a specialized ambulance nurse (EMS clinician) and a specially trained ambulance driver. The EMS clinician is primarily responsible for the EMS care process. The driver assists the clinician in performing diagnostic and therapeutic tasks, has logistical duties, and drives the ambulance. EMS clinicians in the Netherlands are broadly trained. After earning their bachelor’s degree in nursing, most of them completed a nursing specialization in at least one of the following areas of interest: Critical Care, Coronary Care, Hospital Emergency Medicine (all 1 year) or Anesthesiology (2 years). To practice as an EMS clinician, an 8-month specialist training is required after the initial nursing education. Thus, the total training duration for practising the profession covers a minimum of 5 years and 8 months (bachelor 4 years, specialization at least 1 year, ambulance endorsement 8 months). The ambulance driver training takes 7 months and includes training in driving skills and medical assistance tasks. Dutch ambulance personnel work according to national protocols and standards. They are formally supervised by a physician but handle most of the cases autonomously. However, a doctor is available 24 h a day, 7 days a week, for telephone consultation.

### Selection of participants

We invited EMS clinicians who met the following inclusion criteria to participate voluntarily in the study:

- practice EMS care on a daily basis.
- are registered as an EMS professional according to Dutch law.
- signed written informed consent at the time of joining the study.

### Data collection: real-life video observations

To gather the video footage with minimal interference of the real-life clinical process, we equipped the participants with special video glasses (Ricon 1080p, Ivue Camera, Sugar City ID, USA [13]) with an integrated micro-camera and microphone. During their routine work, the EMS clinicians wore these glasses. The camera was manually activated directly after the participant was dispatched to a patient call, enabling recording participants’ pre-arrival activity. The recording was ceased after the final transfer. Thus, the complete deployment was videotaped. At the end of the EMS response, written informed consent from the patient was obtained.

We expanded the core research group with nursing students to carry out the data collection and processing. All nursing students were in the last phase of their bachelor program and had finished a specific Critical Care education trajectory and a dedicated qualitative data gathering and analysis course. These student researchers were assigned to the participating EMS clinicians to assist them in operating the camera and carrying out formal research tasks.

They were added to the ambulance crew and rode along with the ambulance deployments daily during the study. We recorded video footage of 35 real-life EMS deployments of EMS clinicians employed by the same EMS organization, using a non-participant observational approach [12]. In a pilot phase of the research, we used the first 7 of these videos to define a tentative conceptual model of the EMS care process. The remaining 28 videos were used to test and refine this model until we proposed the definitive model.

### Data collection: semi-structured interviews

Subsequently, to evaluate the definitive model’s recognizability and finally validate it, we interviewed 37 EMS-clinicians. We randomly approached EMS clinicians to participate voluntarily. These clinicians were not previously involved in the research’s video-observational part. Student researchers interviewed the participants using a semi-structured approach based on a predefined topic list (see additional file 1).

The interviews were audiotaped by the interviewers and, afterwards, verbatim transcribed.

The participants were allowed to describe their own EMS care process freely. The interviews continued by introducing the defined conceptual model, using the schematic representation (Table 1). We asked the participants to react to it and explicitly asked them about the recognizability of the model.

### Analysis: real-life video observations

In a pilot study, the principal investigator (BD) analyzed the first 7 video-recorded cases using a thematically constant comparative method using open coding, axial coding and selective coding, according to the method described by Strauss and Corbin [14, 15]. The qualitative data analysis software package Atlas.ti (versions 8/9, Cleverbridge AG, Cologne, Germany) was used to facilitate the analysis. We focused on observable diagnostic and therapeutic activities in the care process. In the open coding phase, we coded both the professional diagnostic or therapeutic activities performed and the verbal interaction between team members or between a team member and the patient or relatives.

For example, auscultation of the lungs was *open coded* as “physical examination”. In an iterative cycle of open coding, “physical examination” was divided into two subcodes, “targeted physical examination” and “general physical examination”, depending on the context in which this examination took place. If the examination was executed while exploring the main complaint (e.g., shortness of breath), we classified it as “targeted”. If it was done as a part of a standard general medical examination, we subcoded it “general”.

Using *axial coding*, we merged subcodes into categories. For instance, we combined the subcodes “anticipation of the situation on scene” and “pre-arrival division of workload” into the category code “Pre-Arrival-Preparation”, using a term commonly known in the field.

With *selective coding*, we grouped autonomous subcodes and categories in different EMS care process phases. The unifying factor in these phases was either the timespan where the execution of a specific activity took place or the existence of a shared goal of different activities. For instance, topics related to activities executed during the timespan from “dispatch” to “arrival-on-scene” were grouped in the care process phase: “Start”, while all actions related to mapping the presenting problem were grouped as “Presentation”.

This analysis led to the formulation of a tentative model consisting of several process phases.

The remaining 28 video recorded cases were examined by student researchers for the presumed phases’ presence or absence. They also searched for the defined subcodes and categories. Finally, they looked for the existence of topics that were not recognized before. The student researchers worked in pairs. Each pair focused on a subset of all the phases in the model. Each student of a pair autonomously researched the data for their assigned subset. After that, they compared their findings and discussed different interpretations until they reached a consensus. During this process, the student-researchers were continuously methodologically supported by teacher-researchers or members of the research team. In plenary meetings with the entire research group, each pair reported their findings. The results were combined. With the researched cases, data saturation was reached, and the definitive model was defined (see additional file 3).

### Analysis: real-life video observations

The 37 interview transcripts, again, were thematically analyzed using Atlas.ti software. Student researchers independently coded the transcripts. After that, they discussed differences in pairs until they reached a consensus. The pairs reported to the entire research group.

## Results

### Characteristics of the study and the study subjects

We included 35 clinical cases with a total recorded time of 25.7 h. The clinical situations represented sufficiently the normal EMS workload (Table 1). Twenty participants handled these cases (Table 1). We interviewed 37 other participants (Table 1). In both groups, participants’ diversity in gender, age, and professional experience was sufficient to consider the group as a representative sample of the average EMS working population in the Netherlands [16].

**Table 1:** Characteristics of video recorded clinical situations

	n
chest pain	7
dyspnoea	2
accident	11
neurologic complaints/deficits	5
unconsciousness	3
haemorrhage	3
violent abuse	1
collapse	2
malaise	1
**total**	**35**

**Table 2:** Characteristics of participants video observations

	*n*	*%*	*mean*	*SD*
*gender*				
male	12	60		
female	8	40		
age (y)			44.2	8.4
total experience as an RN (y)			19.9	10.0
experience as an EMS clinician (y)			10.2	6.6

**Table 3:** Characteristicts of interviewees content analysis

	*n*	*%*	*mean*	*SD*
*gender*				
male	26	70		
female	11	30		
age (y)			45.4	9.5
experience as an EMS clinician (y)			14.7	9.3

### Video observed cases

Analysis of the pilot study’s data resulted in the formulation of a preliminary conceptual model of the EMS care process. Further analysis of the remaining data optimized the preliminary conclusions. We distinguished ten care process phases which covered all the observed actions (see additional file 2). The name of each phase describes the relationship of actions performed in that phase. We will describe the most obvious observations per phase.

#### start

Most actions we observed in this phase are related to reading the digitally provided dispatch information. Interpretation of the available information often (but not always) led to the preliminary formulation of possible diagnoses. Sometimes additional information was requested from to dispatch center. In a few cases, the clinician decided beforehand (based on the dispatch information) that a particular treatment had to be given or that transfer to a hospital would be inevitable.

Second most actions that were seen were related to the Pre-Arrival-Preparation (PAP). The preparation varied from donning gloves to drawing up an attack plan and distributing tasks among the crew. The abovementioned actions implied extensive communication between crewmembers.

#### situation

While approaching a scene, in particular, while arriving at an outdoor accident, the EMS crew interpreted the situation and often decided in a split second whether an acute intervention was necessary. Based on the observed peer-to-peer communication, we concluded that the formulation of preliminary diagnoses at this point of the process does exist.

#### presentation

The data revealed that clinicians almost always focus primarily on the patient’s complaint, injury, or health problem during initial contact with a patient.

In this phase, focused questioning and targeted physical examination concerning the presenting problem is being executed. Now and then, a written or verbal hand-over from another healthcare professional is a source of information. We expected in advance that the clinician would, at this stage, try to reveal the exact reason for the call for assistance by questioning the patient. In our data, only a few clinicians have formally asked for this reason.

#### prologue

The Prologue is embedded in the Presentation phase. During the Prologue phase, information about factors leading to and influencing the presenting complaint, injury or health problem is collected by asking questions. Now and then, “Prologue” information is gathered purely by observation, for instance, in the case of (road)accidents.

#### anamnesis

Gradually the focused questioning, as mentioned in the Presentation phase, changes to surveying patients’ general medical condition by gathering general anamnestic information. Often the clinicians questioned the patient about his or her medical history, actual medical condition, medication usage, and known allergies. They occasionally asked consent questions about proposed treatment or transfer or questions about treatment restrictions.

#### assessment

General physical examination is often routinely and standardized performed. Assessment of the respiratory, the circulatory and the neurologic systems is almost standard practice in our researched population. Blood tests (blood glucose, troponin), temperature measurement and pain scores are carried out less frequently. Often the ABCD (Airway, Breathing, Circulation and Disability) method is used to structure the examination of vital stability.

#### reasoning

The synthesis of the gathered clinical, general, and contextual information leading to a provisional conclusion, working diagnosis, or differential diagnosis is considered to be the process of clinical reasoning. The outcome almost always demands a decision. Often the result of this process is an action to be carried out. Our research showed that throughout the complete EMS deployment, topics related to this reasoning process are observable.

It is evident that EMS-clinicians continuously take decisions. Either small: e.g., “is the ECG abnormal or not”, or of greater importance: e.g., “do we have to start with resuscitation or not”. Most decisions we observed were based on rational information and logical interpretation of this information. Clinicians tend to speak out loud and often share their thoughts and considerations with their EMS colleague and sometimes with the patient. Occasionally a decision seemed to be mainly based on intuitive considerations. An example of the latter is the instant intervention executed seconds after an initial encounter.

We found that, without exceptions, somewhere in the deployment, the EMS-clinician took a moment to recapitulate the situation, decided if information was missing, tried to complete the picture and came to a conclusion. This conclusion always led to a definitive resolution: what to do with the patient; “convey or not”, “treat or not”, “hand-over or not”.

#### resolution

The Resolution phase is inseparably linked to the Reasoning phase. In our material, every EMS response was concluded with a final resolution. The clinician literally expressed the defined resolution. Sometimes the resolution was taken in conformity with the patient: a shared decision. Sometimes the clinician took the decision autonomously.

#### treatment

Most of the actions we observed in this phase were related to the (precautionary) placement of an intravenous access line. The administration of intravenous medication came in second place. Most of these medications were analgesics. Official protocols and guidelines supported many treatment actions.

#### transfer

Most patients were conveyed to the hospital. A telephone transfer was consistently carried out before the actual transport. Some patients were left at home with self-care instructions. A few patients were referred to a general practitioner.

### other

The activities that we could not classify under one of the defined phases were assigned the code “Others”. Examples of these codes were: consultation of external resources, consultation with other healthcare professionals, giving information, or providing explanations to the patient or others. Because these activities occurred infrequently in the research materials, we did not position them in the final model.

### Model development

By summarizing our findings, we were able to define the final model. We introduced the acronym “SPART” to name the model and describe its ten phases. One letter in the acronym describes two phases of the process: Start, Situation, Prologue, Presentation, Anamnesis, Assessment, Reasoning, Resolution, Treatment and Transfer.

Table 1 demonstrates the different phases and their related  activities.

**Table 4:** Phases and activities SPART model

	Phase	Activities
S	Start	-Initiation of the EMS deployment. Emergency call-taking and EMS dispatch. -Interpretation of the information provided by the dispatch center (first generation of clinical hypotheses). -Pre-Arrival-Preparation (dividing tasks among the crew, anticipating the expected situation on the scene)
	Situation (at arrival)	-First subjective, and intuitive interpretation of the scene. -Ongoing generation of clinical hypotheses: “a wet read diagnosis.” -Decision whether acute intervention is necessary.
P	Prologue	-Retrospective interpretation of factors leading to and influencing the presenting complaint, injury or health problem -In case of an accident: interpretation of the accident mechanism.
	Presentation (presenting complaint or symptom)	-Indicating the reason for the call for assistance. -Performing focused questioning and targeted physical examination, focused on the primary complaint, injury or health problem.
A	Anamnesis	-Medical history taking. -Inventory of medication and allergies. -Identification of treatment restrictions.
	Assessment	-General physical examination. -Assessment of vitals (ECG, BP, HF, RR, SpO_2_). -Neurologic examination, if applicable. -Taking blood samples, if applicable.
R	Reasoning, recapitulation	-The actual process of gathering, ordering, evaluating, and interpreting clinical information to formulate a working diagnosis and consider differential diagnoses. -A clinical time out to overview the gathered information and detect information deficiencies.
	Resolution	-The (clinical) decision on what to do or do not.
T	Treatment	-Therapy, if possible, and applicable in the pre-hospital setting.-Guided by protocols and guidelines.
	Transfer	-Mandatory to conclude the EMS deployment. -Three possible routes: 1. To the patient self. Clinical therapy or conveyance to the hospital or both are not necessary. Shared decision process. Informed consent. With dedicated attention to patients’ questions, fears and uncertainties. 2. Hand-over to other (professional) care provider (i.e., GP, midwife, mental health care provider). 3. Conveyance and hand-over to a hospital or other care facility. -Evaluation and reflection

### Semi-structured interviews

We tested the final SPART model for its validity and recognizability by conducting a peer content analysis. We hypothesized that the degree of recognition by interviewees would be an indication of the model’s realism and would say something about its validity. The peers were considered experts in the field as they provide EMS care on a daily basis and have a significant amount of professional experience.

It was evident that all participants executed their care process in a more or less similar way. The care process they run through and the description of the different phases of a deployment were highly comparable across the group and matched the phases we defined in the SPART model. The participants described the first action they executed during a callout: reading and interpreting the dispatch data provided to them via the mobile data terminal. The majority took this action together with their colleague, discussing their expectations and dividing up the tasks.

*(participant 19) “Well, then you consult with your colleague about, oh, what to expect, what do we need to think about, what do we need to take with us in terms of equipment?”*

Half of the questioned participants mentioned that they formulated preliminary medical hypotheses based on the available information.

*(participant 10) “Immediately after receiving the dispatcher’s information, I start forming images in my head of the expected situation we are going to find; What are we going to see?”*

After arrival on the scene, one-third of the questionees mentioned a “first impression of the situation”. They gather contextual information to determine whether the situation is safe. Using the so-called Patient Assessment Triangle (PAT), they make a rapid estimate of the patient’s condition and decide whether acute intervention is required. The decision whether there is time or not is mainly driven by experience, intuition, and pattern recognition.

*(participant 8) “Then, the first look at the patient is often based on gut feelings. How is someone breathing? What is the general impression? I say, well, this is not too bad, or this is, we must not dawdle …*”.

After introducing themselves, they commenced with questioning and examining the patient, focusing on the problem he or she presents with. Almost always, at the same time, the colleague crewmember proceeds with hooking up the patient to the monitor to measure vital parameters objectively.

*(participant 7)” …what exactly is going on, in terms of time, what are the symptoms, what kind of pain, how long has it been there, so you gradually gather some more information about the reason why you are there…”.*

As the care process progresses, at a certain point, all the questioned clinicians decided whether to treat or not, whether to convey or not and whether to hand over to another professional or not. Only a few mentioned that this decision is a shared decision taking into account the patient’s wishes.

The care process is, without exception, concluded with a verbal hand-over. Often, they use the SBAR acronym to structure this hand-over.

After the introduction of our SPART model, almost all the participants recognized the phases of the model. The suggested order of most phases was also recognizable. They stated almost unanimously that the model describes reality well but noted that the model is used more flexibly in everyday practice sequencing.

*(participant 10)” The work that we actually do is now very neatly laid out in a diagram.”*

*(participant 13)” Actually, I think it is pretty similar. They just gave it a different name.”*

*(participant 29) “I never realized that you could give a name to this like SPART but eh, I guess that is how we act.”*

*(participant 16)” It is a nice capstone for trainee EMS-nurses.”*

### Model validation

Overseeing the interviews, we concluded that the majority of the interviewees recognized both the phases in the SPART model and the independent content topics (actions) included in the model. Moreover, they see them as relevant. The phases and actions were also seen as sufficiently distinctive from each other in their meaning.

It can also be concluded that the interviewees missed no essential phases or substantive actions. Except for a few participants, all participants found the model useful and helpful, especially for the younger, less experienced colleagues and the colleagues in training.

## Discussion

In this study, we proposed, refined, and validated a descriptive EMS care process model. The SPART model describes the different phases EMS clinicians run through while they fulfil their deployment. Utilizing a focused ethnographic analysis of real-life cases, we were able to construct a model that realistically represents the EMS care process.

Of course, because any model is an approximate reflection of reality, it must be interpreted and used flexibly. The phases described do not always follow each other in the order indicated. They are also regularly skipped or duplicated. However, our data did confirm the presupposition that all phases are completed at least once. Despite these reservations, this research demonstrated that EMS clinicians recognize their care process in the model. We can conclude that the model matches well with the real-life EMS care process.

Referring to Carter and Thomson “paramedic process” [8], this research demonstrated similarities and differences with their model. For example, in their model, a general patient examination is defined as two single phases of patient history (anamnesis) and physical examination (assessment). We noticed that in real life, each of these phases is divided into two distinctive subphases. We observed that in practice, patient examination is executed in four distinguishable parts. All observed and questioned participants first focused on the presenting problem (Presentation and Prologue) before examining the patient’s general medical status (Anamnesis and Assessment). This phenomenon matches humans’ natural behaviour in that their attention is drawn by the most striking phenomenon in a new situation. This effect is known as the focusing effect [17]. It became evident that the researched clinicians used the Presentation and Prologue phases mainly for developing a working diagnosis, while the Anamnesis and Assessment phases help to determine differential diagnostic considerations. According to Cutrer et al. [18], the formation of early hypotheses and a hypothesis-driven history and physical examination, as we mainly observed in the presentation phase, facilitates efficient data gathering by asking pointed questions. In a certain sense, Carter and Thomson’s “First impressions” phase could be translated into our “Situation” phase. Although this phase is difficult to interpret since it is often run through in silence, we found indirect evidence of its existence. Doorstep-interpretation of the situation can trigger direct intervention. Application of oxygen, e.g., as one of the first actions executed, is considered to be an indirect indication for the existence of a “Situation” phase.

By developing a descriptive EMS care model, we aimed to facilitate identifying factors that influence the clinical reasoning process. We believe that with the development of the SPART model, we have fully mapped the EMS care process. Understanding the care process and naming its components is the first step in identifying weaknesses in it. Further research has to be carried out to find points where the process can be improved. Although more work has to be done, we consider the model already a useful cognitive aid in EMS personnel’s clinical daily reasoning process. It provides an overall structure that reaches beyond the commonly used instruments like, for instance, the acronym ABCDE. The ABCDE mnemonic focuses solely on the information concerning vital functions. SPART takes into account all possible sources of information, both rational objective and intuitive subjective. Moreover, the model covers the entire care process and not just a limited part.

The first two phases of the model, Start and Situation, provide mainly intuitive information, while the remaining phases require a more analytic approach to collect information. This makes the model a practical interpretation of previously formulated concepts in which the dual-process theory [19–22] forms the base of the clinical reasoning process. The model facilitates the intuitive pathway [23] of clinical decision making.

We also suppose SPART provides structure in clinical reporting and could be used as a hand-over instrument. In addition to the model’s clinical benefits, it can be used as a theoretical framework for other EMS research. The model can also be used in an educational context and could help trainees and novice professionals understand professional practice better.

Finally, it could be used for meta-cognitive purposes. That is, it could provide a backbone for self-reflection of EMS providers.

## Conclusions

**Fig. 1:**
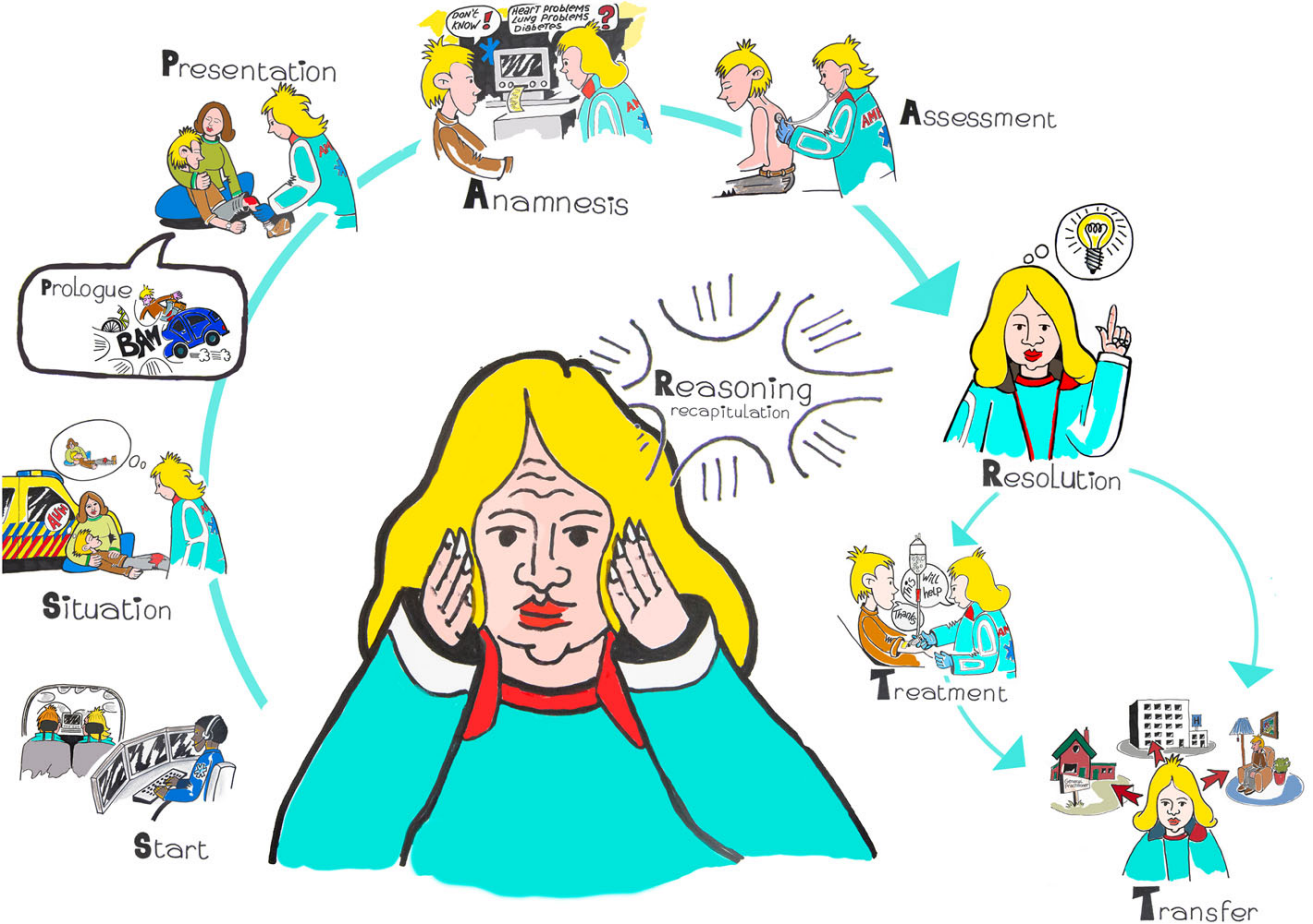
The Spart model (drawing by Anne Woudwijk, ©Bert Dercksen)

We hypothesized that understanding the structure of the EMS care process would be helpful in identifying and addressing issues that impede clinical reasoning in the pre-hospital setting. The EMS care process is complex and so far, only broadly described. Therefore, based on ethnographic observations, we constructed a descriptive model of this process, the SPART model. The SPART model fully and accurately describes the ambulance care process and provides tools to study and optimize the clinical reasoning that is woven into it. In addition to its intended purposes, the model appears to be a useful cognitive tool in practical clinical reasoning and could be useful in medical education, medical reporting, and further EMS-related (cognitive) research. Figure 1 visually depicts the SPART model.

### Limitations

The research was executed in the Netherlands. The research subjects were Dutch EMS clinicians. Because of differences in EMS systems worldwide, extrapolation of the results to other countries and other EMS systems must be done with prudence.

Also, the research population was a self-selected sample which could bias the results.

## Supplementary Information

**Additional file 1:.** Topic list questionary peer content validation.

**Additional file 2:.** Code network SPART codes.

**Additional file 3:.** “Groundedness” and frequency tables SPART codes.

## Data Availability

The video and audio datasets analyzed as part of this study are not publicly available due to the identifiability of individuals and patients. Anonymized datasets are available from the corresponding author upon reasonable request.

## Abbreviations

EMS: Emergency Medical Services
SPART: Start, Situation, Prologue, Presentation, Anamnesis, Assessment, Reasoning, Resolution, Treatment and Transfer
RN: Registered Nurse
PAP: Pre Arrival Preparation
ABCD(E): Airway, Breathing, Circulation, Disability (and Environment)
ECG: Electrocardiogram
BP: Blood Pressure
HF: Heart Frequency
RR: Respiratory Rate
SpO_2_: Oxygen saturation measured with pulse oximetry
GP: General Practitioner
PAT: Patient Assessment Triangle
SBAR: Situation, Background, Assessment and Recommendation

## References

[1] Graber ML (2013). The incidence of diagnostic error in medicine. BMJ Qual Saf.

[2] The CP (2003). Importance of Cognitive Errors in Diagnosis and Strategies to Minimize Them. Acad Med.

[3] Croskerry P (2009). Clinical cognition and diagnostic error: applications of a dual process model of reasoning. Adv Health Sci Educ.

[4] Norman G (2009). Dual processing and diagnostic errors. Adv Health Sci Educ.

[5] Higgs J, Jones M, Loftus S, Christensen N (2008). Clinical reasoning in the health professions.

[6] Graber ML, Franklin N, Gordon R (2005). Diagnostic error in internal medicine. Arch Intern Med.

[7] Andersson U, Maurin Söderholm H, Wireklint Sundström B, Andersson Hagiwara M, Andersson H (2019). Clinical reasoning in the emergency medical services: an integrative review. Scand J Trauma Resusc Emerg Med.

[8] Carter H, Thompson J (2015). Defining the paramedic process. Aust J Prim Health.

[9] Toney-Butler TJ, Thayer JM (2019). Nursing process [internet].

[10] Knoblauch H (2005). Focused ethnography. Forum Qual Soc Res.

[11] Cruz EV, Higginbottom G (2013). The use of focused ethnography in nursing research. Nurse Res.

[12] Nonparticipant Observation. In: Encyclopedia of Case Study Research [Internet]. 2455 Teller Road, Thousand Oaks California 91320 United States: SAGE Publications, Inc.; 2010 [cited 2020 Nov 20]. Available from: http://methods.sagepub.com/reference/encyc-of-case-study-research/n229.xml

[13] iVUE Camera - HD Action Camera Glasses [Internet]. iVUE Camera - HD Action Camera Glasses. [cited 2020 Jun 18]. Available from: https://ivuecamera.com/

[14] Corbin J, Strauss A (1990). Grounded theory research: procedures, canons, and evaluative criteria. Qual Sociol.

[15] Strauss A, Corbin J. Basics of Qualitative Research, Techniques and Procedures for developing Grounded Theory. 4th ed: Sage Publications Inc; 2015. p. 456.

[16] Facts and Figures 2019 National Ambulancecare organisation the Netherlands (AZN) [Internet]. [cited 2021 Jan 7]. Available from: https://www.ambulancezorg.nl/themas/sectorkompas-ambulancezorg/sectorkompas-en-tabellenboeken-%28vanaf-2016%29

[17] Schkade DA, Kahneman D (1998). Does Living in California Make People Happy? A Focusing Illusion in Judgments of Life Satisfaction. Psychol Sci.

[18] Cutrer WB, Sullivan WM, Fleming AE (2013). Educational Strategies for Improving Clinical Reasoning. Curr Probl Pediatr Adolesc Health Care.

[19] Pelaccia T, Tardif J, Triby E, Charlin B (2011). An analysis of clinical reasoning through a recent and comprehensive approach: the dual-process theory. Med Educ Online.

[20] Brush JE, Sherbino J, Norman GR (2017). How Expert Clinicians Intuitively Recognize a Medical Diagnosis. Am J Med.

[21] Diederich A, Trueblood JS (2018). A dynamic dual process model of risky decision making. Psychol Rev.

[22] Stolper E, Van de Wiel M, Van Royen P, Van Bokhoven M, Van der Weijden T, Dinant GJ (2011). Gut Feelings as a Third Track in General Practitioners? Diagnostic Reasoning. J Gen Intern Med.

[23] Croskerry PA (2009). A universal model of diagnostic reasoning. Acad Med.

