# Supplementary material for: Qualitative development and content validation of the “SPART” model; a focused ethnography study of observable diagnostic and therapeutic activities in the emergency medical services care process
Source: BMC Emerg Med. 2021 Nov 13;21:135. doi: 10.1186/s12873-021-00526-z (PMC8590330; doi:10.1186/s12873-021-00526-z)
Supplement: Supplementary file 1 — Additional file 1:. Topic list questionary peer content validation. [file 12873_2021_526_MOESM1_ESM.docx]

**Qualitative development and content validation of the “SPART” model; additional file 1**

Topic list questionary peer content validation

*Introduction*

Objective’s questionary:

1. To investigate whether, and if so, in what way, the participant structures his own EMS care process.

2. To investigate whether, and if so, in what way, the participant in their clinical reasoning process uses a (more or less) fixed structure to reach a clinical decision.

3. To investigate whether, and if so, to what extent, the participant recognizes the demonstrated SPART model as a representation of his daily workflow when practising EMS care.

Demographic questions:

- gender

- age (y)

- experience as an EMS clinician (y)

Questions:

-Could you describe your own EMS care process?

-Could you distinguish steps, phases, or actions in your process?

-Could you describe your clinical reasoning process and the formulation of a clinical

decision?

*Demonstration of the SPART model*

Questions continued:

- Could you reflect on this SPART model and compare it to the structure you use in your own EMS care process?

-As you review the model, can you comment on the defined phases considering their accuracy, suggested sequence, and completeness?

- To what extent do you think this model could contribute to the critical and clinical reasoning process?

*Closure*

Questions continued:

- Do you have any general comments or remarks?

- Thank you for your time.
