# Supplementary material for: Qualitative development and content validation of the “SPART” model; a focused ethnography study of observable diagnostic and therapeutic activities in the emergency medical services care process
Source: BMC Emerg Med. 2021 Nov 13;21:135. doi: 10.1186/s12873-021-00526-z (PMC8590330; doi:10.1186/s12873-021-00526-z)
Supplement: Supplementary file 2 — Additional file 2:. Code network SPART codes. [file 12873_2021_526_MOESM2_ESM.docx]

**Qualitative development and content validation of the “SPART” model; additional file 2**

Code network SPART codes

**
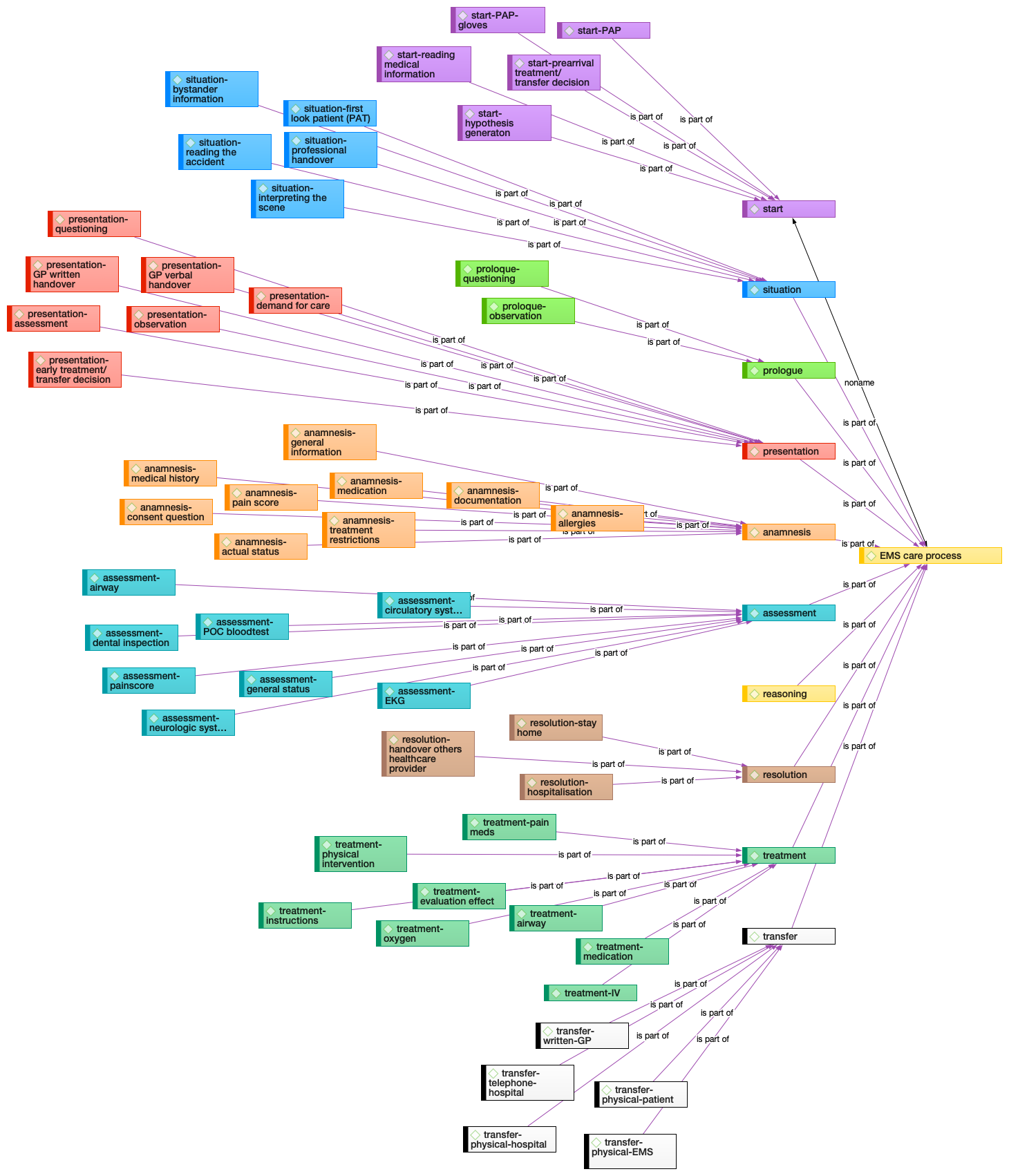
**
